# Optimizing Outcomes in Mismatched Unrelated Donor Allogeneic Transplantation: Post-Transplant Cyclophosphamide’s Dual Impact on Graft versus Host Disease Incidence and Overall Survival: Retrospective Analysis on Behalf of Polish Adult Leukemia Group

**DOI:** 10.3390/jcm13123569

**Published:** 2024-06-18

**Authors:** Jarosław Dybko, Małgorzata Sobczyk-Kruszelnicka, Alicja Sadowska-Klasa, Agnieszka Piekarska, Sebastian Makuch, Siddarth Agrawal, Krzysztof Dudek, Ugo Giordano, Sebastian Giebel, Lidia Gil

**Affiliations:** 1 Department of Hematology and Cellular Transplantation, Lower Silesian Oncology Center, 53-413 Wroclaw, Poland; 2 Department of Oncology and Hematology, Faculty of Medicine, Wroclaw University of Science and Technology, 58-376 Wroclaw, Poland; 3 Department of Bone Marrow Transplantation and Oncohematology, Maria Sklodowska-Curie National Research Institute of Oncology, Gliwice Branch, 44-102 Gliwice, Poland; malgorzata.sobczyk-kruszelnicka@io.gliwice.pl (M.S.-K.); sebastian.giebel@io.gliwice.pl (S.G.); 4 Department of Hematology and Transplantology, Medical University of Gdansk, 80-210 Gdansk, Poland; a.sadowska@gumed.edu.pl (A.S.-K.); agnieszka.piekarska@gumed.edu.pl (A.P.); 5 Department of Clinical and Experimental Pathology, Wroclaw Medical University, 50-368 Wroclaw, Poland; sebastian.mk21@gmail.com; 6 Department and Clinic of Internal Medicine, Occupational Diseases, Hypertension and Clinical Oncology, Wroclaw Medical University, 50-556 Wroclaw, Poland; siddarth@agrawal.pl; 7 Faculty of Mechanical Engineering, Wroclaw University of Science and Technology, Wybrzeze Wyspianskiego 27, 50-370 Wroclaw, Poland; krzysztof.dudek@pwr.edu.pl; 8 Department and Clinic of Endocrinology, Diabetes and Isotope Therapy, 50-367 Wroclaw, Poland; ugogiordano1@gmail.com; 9 Department of Hematology and Bone Marrow Transplantation, Poznan University of Medical Sciences, 60-569 Poznan, Poland; lidia.gil@skpp.edu.pl

**Keywords:** MMUD-HSCT, PTCy, GvHD, prophylaxis, transplantation, survival

## Abstract

Allogeneic hematopoietic cell transplantation (allo-HSCT) stands as an effective treatment method for various hematologic malignancies. However, graft-versus-host disease (GvHD), an intricate immunological phenomenon where donor immune cells target recipient tissues, remains a significant challenge, particularly in mismatched unrelated donors (MMUD). Post-transplant cyclophosphamide (PTCy) has emerged as a promising immunosuppressive strategy, revolutionizing haploidentical transplantation and demonstrating promise in MMUD settings. **Background/Objectives**: This study aimed to evaluate the impact of PTCy on MMUD allo-HSCT outcomes, specifically its effects on GvHD incidence and overall survival, compared to anthitymocyte globulin (ATG). **Methods**: One hundred seventy-four patients were classified into three groups based on the type of transplantation: PTCy-haplo (114/174; 65.5%), PTCy-MMUD (23/174; 13.2%), and ATG-MMUD (37/174; 21.2%). **Results**: Our findings showed that PTCy-MMUD significantly reduced acute GvHD occurrence compared to PTCy-haplo and ATG-MMUD approaches (*p* = 0.006). The delayed onset of acute GvHD in the PTCy-MMUD group suggests a more controlled immune reconstitution, contributing to the lower incidence. Importantly, PTCy-MMUD exhibited enhanced five-year overall survival rates, aligning with the notion that reduced GvHD correlates with improved patient outcomes (*p* = 0.032). **Conclusions**: We believe that this study contributes valuable insights into PTCy-MMUD’s management, underscoring its potential to significantly reduce GvHD incidence and enhance survival outcomes. Although further investigations and clinical trials are warranted, this research underscores the promising role of PTCy-based GvHD prophylaxis in improving MMUD allo-HCT success.

## 1. Introduction

Allo-HCT is a curative treatment option for acute leukemias and many other hematologic malignancies [1]. However, GvHD, a complex immunological phenomenon where donor immune cells mistake recipient tissues for foreign and mount an immune attack, frequently hinders the success of allo-HCT. GvHD remains an enormous cause of morbidity and mortality following allo-HCT, specifically in conditions wherein the donor and recipient are mismatched in phrases of human leukocyte antigens (HLA), including in MMUD transplantation [2].

To mitigate the hazard of GvHD and improve transplant consequences, novel regimens for immunosuppression have been explored [3,4]. PTCy has emerged as a promising strategy in recent years. Although PTCy has brought about a transformative shift in the landscape of haplo-HCT [5,6], recent investigations have also unveiled promising results in the context of MMUD-HCT with PTCy utilization [7,8,9]. Moreover, studies have shown that CMV reactivation rates can differ between transplantation protocols that use PTCy-haplo and PTCy-MMUD approaches [7,8,9]. The precise rates and outcomes may fluctuate depending on various factors, such as the demographic of the patient, the conditioning regimen administered, the nature of the donor, and the comprehensive management of CMV prophylaxis and therapy.

According to recommendations formulated by an international panel of experts [10], it is strongly advised to include anti-thymocyte globulin (ATG) administration as a component of the myeloablative conditioning (MAC) regimen before performing bone marrow (BM) or peripheral blood stem cell (PBSC) allo-HCT from either a matched unrelated donor (MUD) or an MMUD, with the purpose of preventing GvHD. Limited evidence also supports the utilization of ATG prior to PBSC allo-HSCT from a matched related donor (MRD). In situations involving reduced intensity or nonmyeloablative conditioning (RIC/NMA) regimens, which carry an elevated risk of relapse, ATG has shown effectiveness in averting both acute GvHD (aGvHD) and chronic GvHD (cGvHD) [10].

More recently, the European Group for Bone and Marrow Transplantation (EBMT) has published updated recommendations. According to these guidelines, ATG is presently recommended for use in MRD allo-HCT. In cases of MMUD and MUD donors, either PTCy or ATG are considered viable options for GvHD prevention [11]. PTCy is established as the standard approach in haploidentical allo-HCT, even in MMUD 4/8 to 7/8 transplants, due to its ability to achieve low rates of severe aGvHD, cGvHD, and non-relapse mortality (NRM). PTCy, when used as a single agent, has demonstrated efficacy in MRD/MUD bone marrow transplantation (BMT) [12,13]. However, in the context of MRD/MUD MAC, BMT and PTCy used in isolation did not exhibit superiority compared to the combination of Tacrolimus (Tac) and methotrexate (MTX) [14]. For MRD/MUD RIC peripheral blood stem cell transplantation (PBSCT), PTCy as a single agent was deemed unsafe, in contrast to PTCy when combined with Tac and mycophenolate mofetil (MMF), which are regarded as the standard regimen for GvHD prophylaxis in this scenario [3,15,16,17,18].

PTCy is effective in transplant settings due to several mechanisms. Primarily, it disrupts the function of rapidly dividing alloreactive donor T-cells, which are cells that could potentially attack the recipient’s body. It also encourages the growth of regulatory T-cells (Tregs), which play a role in immune system moderation and help prevent an overreaction of the immune system. Additionally, PTCy spares non-alloreactive T-cells, which are crucial for fighting tumors and infections [19,20]. In the context of alloreactive T-cells, there’s a difference based on whether the donor and recipient are HLA-matched or mismatched. HLA, or human leukocyte antigen, is a key factor in the immune system’s ability to recognize cells as self or non-self. In HLA-mismatched transplants, alloreactive donor T-cells aggressively target the recipient’s cells, recognizing them as foreign due to major histocompatibility differences. Conversely, in HLA-matched transplants, these reactions are less intense as donor T-cells only recognize minor antigens and thus proliferate less [19,20]. Despite these benefits, there is ongoing debate about the effectiveness of PTCy in HLA-matched allo-HCT settings. This is because the dynamics of T-cell activity differ depending on the HLA compatibility, influencing how well PTCy can modulate immune responses in these scenarios.

This retrospective study aims to analyze the impact of PTCy in the context of MMUD allo-HCT and its impact on the incidence of GvHD and overall survival by evaluating the available clinical evidence.

## 2. Materials and Methods

### 2.1. Study Population

In this retrospective analysis (with pre-defined groups), the study group consisted of 174 patients (83 males and 91 females), of which 145 were aged less than 60 years old (145/174; 83.3%) transplanted between 2015 and 2019 in three different Polish Centres from haploidentical or 9/10 MMUDs with mobilized peripheral blood as the only source of stem cells. Most of them were diagnosed with acute myeloid leukemia and myelodysplastic syndrome (83/174; 47.7%). Other diagnoses include lymphomas and myeloma (44/174; 25.3%), acute lymphoblastic leukemia (ALL) (27/174; 15.5%), osteomyelofibrosis (OMF), chronic myeloid leukemia (CML), and others (20/174; 11.5%). The study group was divided into three groups: PTCy-haplo (*n* = 114), PTCy-MMUD (*n* = 23), and ATG-MMUD (*n* = 37). The estimated median follow-up time for patients alive at the end of the study was 8.5 months. (from 3.0 to 83.6). Full patients characteristics is contained in Table 1.

### 2.2. Conditioning Regimen

The proportion of patients conditioned with a MAC (116/174; 66.6%) was significantly higher than either RIC or NMA regimens (36/174; 20.7% and 22/174, 12.6% respectively. Within the analyzed group, MAC conditioning was applied in 53.5% of cases (61/114) for PTCy-haplo, 100% (23/23) for PTCy-MMUD, and 86.5% (32/37) for ATG-MMUD. MAC was traditionally defined as conditioning with TBI ≥ 5 Gy in one single dose or TBI ≥ 8 Gy in a fractioned dose or one of the following chemotherapy agents: busulfan ≥ 8 mg/kg b.w. (or intravenous equivalent), Treosulfan ≥ 10 mg/m^2^, melphalan ≥ 140 mg/m^2^, or Thiotepa ≥ 10 mg/kg b.w. NMA regimens were mostly fludarabine based with TBI ≤ 2 Gy or TLI or lower doses of cyclophosphamide [21]. The remaining protocols were classified as RIC.

### 2.3. GvHD Prophylaxis

GvHD prophylaxis in the PTCy group consisted of cyclophosphamide at a dose of 50 mg/kg b.w/d. given on days +3 and +4, followed by Tac and MMF (110/145; 75.9%). The ATG group was given either ATG-Grafalon^®^ in an average total dose of 20 mg/kg b.w. or Thymoglobuline^®^ in a total dose of 4.5 mg/kg b.w supported by conventional cyclosporine and methotrexate prophylaxis (35/145; 24.1%). For acute GvHD, classical Glucksberg criteria [19] with the MAGIC consortium update [20] were used, and for chronic GvHD, the NIH criteria [21].

### 2.4. Outcomes

The main objective of this retrospective analysis was to compare the impact of PTCy and ATG, as well as different donor settings, on the occurrence of acute and chronic GvHD. The secondary outcomes included CMV reactivations and survival.

### 2.5. Statistical Methods

For quantitative features, the compliance of their distribution with the normal distribution was checked. Agreement was assessed using Shapiro-Wilk’s test. For quantitative variables, mean values (M), standard deviations (SD), medians (Me), lower (Q1) and upper quartiles (Q3), and range of variability (Min) and (Max) were calculated. The significance of differences in mean values in two groups for features with a significantly non-normal distribution or with heterogeneous variances was checked using the non-parametric Mann-Whitney U test. Qualitative variables (measured on nominal or ordinal scales) were presented in contingency tables as numbers (*n*) and proportions (%), and the chi-square test or Fisher’s exact test was used to assess the strength of the relationship between two variables.

The Kaplan–Meier method was used to estimate patients’ probability of survival. The incidence of aGvHD and cGvHD and overall survival after transplantation were used as an objective parameter to assess the treatment’s efficacy. Multivariate logistic regression analysis was used to assess the impact of the analyzed parameters on the occurrence of GvHD. Its results are presented in Table 2 and Table 3. Threshold values for donor age were determined by ROC curve analysis. All analyses were performed using the statistical software package Statistica v.13.3 (TIBCO Software Inc. Palo Alto, CA, USA). A *p*-value of <0.05 was considered to be statistically significant.

## 3. Results

In this study, we classified 174 patients into three groups based on the type of transplantation: PTCy-haplo (114/174; 65.5%), PTCy-MMUD (23/174; 13.2%), and ATG-MMUD (37/174; 21.2%).

While analyzing the groups, we observed a substantial reduction in the occurrence of acute GvHD in recipients who received PTCy as part of their GvHD prophylaxis regimen. Notably, acute GvHD was markedly lower in the PTCy-MMUD group (21.7%) compared to the PTCy-haplo (35.1%) and ATG-MMUD (59.5%) groups. Moreover, the median time to onset of acute GvHD was significantly delayed in the PTCy-MMUD group (27 days) compared to the PTCy-haplo (41 days) and ATG-MMUD (18 days) groups.

Our study underscores the profound influence of PTCy on overall survival rates. The five-year overall survival probability was significantly enhanced in the PTCy-MMUD group (73.4%) compared to the PTCy-haplo (45.3%) and ATG-MMUD (33.4%) groups. Notably, while cumulative nonrelapse mortality did not exhibit significant differences between groups, the progression-free survival rate displayed remarkable disparities, further highlighting the potential benefits of PTCy in enhancing treatment outcomes.

### 3.1. Survival

**Figure 1:**
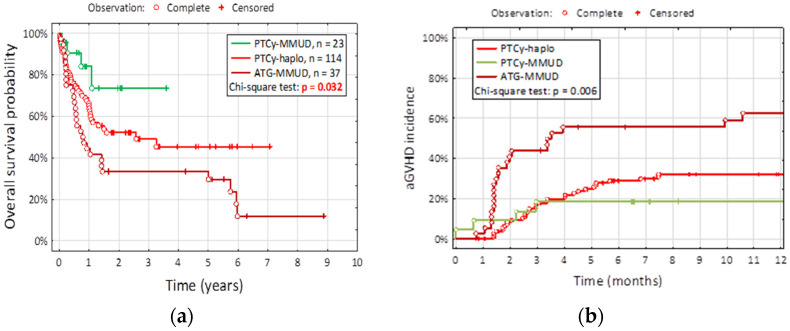

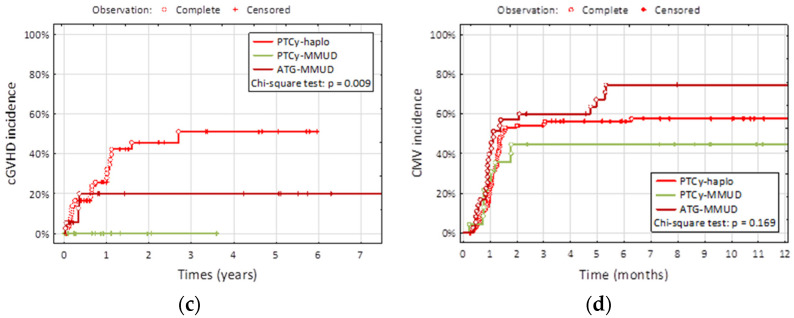
Kaplan–Meier curves for the following: (**a**) Overall survival; (**b**) aGvHD incidence; (**c**) cGvHD incidence; (**d**) CMV incidence.

We compared the overall survival (OS) probability in PTCy-MMUD, PTCy-haplo, and ATG-MMUD groups (3 years OS—73.6% vs. 49.1% vs. 33.4%, respectively, *p* = 0.032) and presented the results in Figure 1a. The three-year OS probability in the ATG-MMUD group is significantly lower than in the PTCy-haplo and PTCy-MMUD groups. The difference in OS between patients in the PTCy-MMUD and ATG-MMUD groups is statistically significant (*p* = 0.008) also for the 2- and 3-year period, with the former guaranteeing more favorable outcomes. A similar result can be observed when the PTCy-MMUD and PTCy-haplo cohorts are compared, but it did not yield statistical significance (*p* > 0.05).

### 3.2. GvHD

Of a total of 174 patients, 67 had acute GvHD (67/174, 38.5%). Within the analyzed groups, acute GvHD occurred among 40/114; 35.1% of PTCy-haplo, 5/23; 21.7% of PTCy-MMUD, and 22/37; 59.5% of ATG-MMUD (*p* = 0.006), as shown in Figure 1b. In the ATG-MMUD group, the number of patients with aGvHD grade 3–4 was significantly higher compared to the PTCy-haplo group (21.6% vs. 5.3%, *p* = 0.009), similarly compared to the PTCy-MMUD (21.6% vs. 8.7%, *p* = 0.04), there was no significant difference for aGvHD grades 3–4 between PTCy-MMUD and PTCy-haplo as well as for grades 1–2 between the three groups (ATG-MMUD vs. PTCy-MMUD—37.8% vs. 13.0%, *p* = 0.114; PTCy-MMUD vs. PTCy-haplo (13.0% vs. 29.8%, *p* = 0.196; PTCy-haplo vs. ATG-MMUD—29.8% vs. 37.8%, *p* = 0.364). Furthermore, the median time of onset of aGvHD was 41 days vs. 27 days vs. 18 days, respectively (*p* = 0.001). When considering chronic GvHD it was observed among 28 patients in the PTCy-haplo group (28/114, 24.6%). None out of 23 PTCy-MMUD group and 4/37—10.8% displayed signs of cGvHD (*p* = 0.009). The detailed results can be found in Figure 1c.

In multivariate analysis, the risk factors of aGvHD were ATG-MMUD and donor age. The risk of developing aGvHD among patients in the ATG-MMUD group is over four times higher compared to the other cohorts (OR = 4.24). Similarly, in the group of patients whose donor age was equal to or greater than 31, the risk of aGvHD is also over four times higher compared to patients whose donor was younger (OR = 4.34). For cGvHD, PTCy-haplo and donor age appeared to be risk factors for its development. The risk of cGvHD among patients in the PTCy-haplo group is more than three and a half times higher compared to the others (OR = 3.73); in the group of patients whose donor age was equal to or greater than 42, the risk of cGvHD is also more than three and a half times higher compared to patients whose donor was younger (OR = 3.54).

### 3.3. CMV Reactivation

All patients were transplanted in the pre-letermovir era. There were no differences in CMV prophylaxis between groups as well as in CMV disease. The ATG-MMUD group displayed a lower percentage of CMV reactivation (29.7%); however, the difference between the three analyzed groups came out not significant (*p* = 0.169), as shown in Figure 1d, but compared to the number of CMV copies before and after antiviral treatment (if CMV reactivation was detected) we found significant differences (≥250 copies before treatment—54/114; 47.4% (PTCy-haplo) vs. 8/23; 34.8% (PTCy-MMUD) vs. 25/37; 67.6% (ATG-MMUD) *p* = 0.03; after treatment ≥ 250 copies 13/114; 11.4% vs. 0/23; 0/23; 0.0%, vs. 11/37; 29.7%; *p* = 0.011, respectively. Ganciclovir was the first treatment choice in the ATG-MMUD group; valganciclovir is in the PTCy-MMUD group with no prevalence in the PTCy-haplo group (*p* < 0.001).

### 3.4. Neutrophil Recovery

Neutrophil recovery (absolute neutrophil count (ANC) > 0.5 × 10^9^/L) was significantly delayed in both PTCy groups. The median time after transplantation with ANC > 0.5 × 10^9^/L was 21 days for both PTCy groups and 15 days for the ATG-MMUD group (*p* < 0.001).

## 4. Discussion

This study found that the PTCy-MMUD setting significantly reduced the occurrence of acute GvHD compared to PTCy-haplo and ATG-MMUD groups, which predominantly used MAC conditioning and PBSC as the cell source. The delayed onset of acute GvHD in the PTCy-MMUD group suggests more controlled and gradual immune reconstitution, contributing to the lower GvHD incidence. This underscores the critical role of PTCy in modulating post-transplant immune responses. Importantly, the PTCy-MMUD group showed significantly higher three-year overall survival rates compared to other groups, aligning with the concept that reduced GvHD leads to better patient outcomes. While cumulative non-relapse mortality did not differ significantly, the substantial differences in progression-free survival rates highlight the potential of PTCy in enhancing treatment effectiveness. Acute GvHD was significantly lower in the PTCy-MMUD group, indicating PTCy’s potential impact on early immune responses, and the absence of chronic GvHD suggests a protective effect against long-term immune complications. Delayed neutrophil recovery in both PTCy groups, reported previously [22,23,24], is attributed to the immunosuppressive effects of PTCy and did not affect overall survival, emphasizing the importance of monitoring post-transplant immune reconstitution.

The survival analysis revealed clear advantages of PTCy-MMUD in overall and progression-free survival compared to PTCy-haplo and ATG-MMUD groups, suggesting that PTCy-MMUD not only reduces GvHD but also positively impacts disease relapse and other factors influencing long-term survival. These findings align with a study by Battipaglia et al. [25], comparing PTCy and ATG GvHD prophylaxis for AML patients transplanted from 9/10 MMUDs, showing PTCy associated with lower grade III-IV aGvHD and higher leukemia-free survival. Similar results were obtained by Nykolyszyn et al. [26], with lower acute GvHD, non-relapse mortality, and relapse rates. To note, another Battipaglia study showed similar outcomes for ATG and PTCy in patients with AML undergoing alloHCT from MRD [27]. Similarly, a German study did not validate PTCy superiority in MRD, MUD, and MMUD settings [28]. Batipaglia et al. [29] also found lower leukemia-free survival and OS rates in PTCy-haplo compared to PTCy-9/10-MMUD settings, with no notable differences in acute and chronic GvHD. Corresponding to our research, a retrospective study comparing PTCy and thymoglobulin for GvHD prophylaxis in MMUD transplants showed PTCy recipients had prolonged engraftment times but lower acute and chronic GvHD rates at one year [8]. Multivariable analyses indicated no significant differences in survival, relapse, and GvHD-free relapse-free survival between the groups, but thymoglobulin was associated with higher incidences of acute and chronic GvHD and non-relapse mortality. Another study by Jimenez et al. demonstrated the positive impact of PTCy-based prophylaxis compared to Tac/MTX/ATG-based prophylaxis in MMUD allo-HSCT recipients, with higher one-year GvHD-free, relapse-free survival, and overall survival rates in the PTCy group [30] and these results are consistent with ours. A retrospective analysis by Jorge et al. supports our findings: no differences were noted between PTCy-MMUD and ATG-MUD in the 100-day cumulative incidence of acute GVHD grades II to IV and III to IV. Additionally, there were no differences between the groups in terms of non-relapse mortality and relapse rate [31]. Similar conclusions were drawn from the meta-analysis by Tang et al. within the context of allo-HCT from unrelated donors (both MUD and MMUD), using PTCy prophylaxis, showed reduced occurrence of grade II–IV and grade III–IV acute GvHD, diminished non-relapse mortality, and superior overall survival compared to ATG-based regimens. Conversely, chronic GvHD and relapse incidence were comparable in both groups [32]. The influence of donor age on GvHD occurrence has been analyzed, showing that younger donors are associated with a lower risk of both acute and chronic GvHD, which our results confirmed [33,34,35].

A recent EBMT database study by Penack et al. [36] analyzed outcomes of ATG versus PTCy prophylaxis in adult patients receiving their first peripheral blood alloSCT from a 9/10 antigen-matched MMUD, showing lower non-relapse mortality and higher overall survival in the PTCy group. Our study’s OS results are consistent with these findings.

In our study, we did not find significant differences in CMV reactivation rates between groups, which is similar to other reports [8,10,31,32,37]. Some studies confirm lower CMV reactivation rates in PTCy versus ATG groups [7,9,38], although results can vary depending on the MUD/MMUD setting and conditioning details. Conversely, a large CIBMTR study indicated that PTCy might be associated with an increased risk of CMV infection [39]. Mikulska et al. [40] highlighted the importance of haploidentical donors, rather than PTCy itself, as a risk factor for viral infections, including CMV. Despite contradictory data, the risk of CMV reactivations post-allo-HSCT seems related to the type of donor. In the context of MMUD allo-HSCT, individuals receiving ATG-based GvHD prophylaxis showed a tendency toward more CMV reactivations and clinically significant CMV infections (CS-CMVis) compared to those receiving PTCy [8,31], with one study even finding statistical significance [7]. Other studies reported lower occurrences of CMV reactivations and CS-CMVis with PTCy compared to ATG, particularly when most or all donors were MUDs [3,33,34,35,39]. While CMV remains a serious infectious complication of allo-HSCT, it does not appear to trigger aGvHD in the PTCy-MMUD setting and does not reduce overall survival rates compared to ATG-MMUD, as shown in several cited studies.

## 5. Conclusions

Our study shows a clear advantage of PTCy-MMUD in overall survival compared to PTCy-haplo and ATG-MMUD groups, suggesting that PTCy-MMUD not only reduces GvHD but also positively impacts disease relapse and other factors influencing long-term survival. Our findings align with previous studies, but we emphasize the specific benefits of PTCy in an MMUD setting in terms of survival outcomes, contrasting with some studies that did not find significant differences between these groups. This adds to the growing evidence that PTCy is an effective GvHD prophylaxis, particularly in the MMUD transplant setting. Considering these findings, the preferred approach for preventing GvHD in MMUD-HCT may be PTCy along with MMF and a calcineurin inhibitor (CNI). However, further investigations and clinical trials are warranted to confirm and extend these findings, potentially refining treatment protocols and improving patient outcomes in the realm of allo-HSCT.

## Figures and Tables

**Table 1:** The characteristics of patients receiving different types of GvHD prophylaxis, including PTCy-haplo, PTCy-MMUD, and ATG-MMUD, with statistically significant variables (in red: *p* < 0.005).

	PTCy-haplo *n* = 114	PTCy-MMUD *n* = 23	ATG-MMUD *n* = 37	*p*-Value
Patient age, years, Me (IQR)	44 (31–56)	54 (44–60)	45 (35–53)	0.101
Sex, *n* (%)				0.628
Male	52 (45.6%)	13 (56.5%)	18 (48.6%)	
Female	62 (54.4%)	10 (43.5%)	19 (51.4%)	
Diagnosis, *n* (%)				0.004
AML + MDS	56 (49.1%)	12 (52.2%)	15 (40.5%)	
ALL	18 (15.8%)	2 (8.7%)	7 (18.9%)	
HL + NHL + MM	32 (28.1%)	1 (4.3%)	11 (29.7%)	
OMF, CML, SAA et al.	8 (7.0%)	8 (34.8%)	4 (10.8%)	
Median donor age, years Me (IQR)	36 (28–47)	33 (25–39)	31 (23–40)	0.027
Donor age, *n* (%)				0.021
<40 years	64 (56.1%)	19 (82.6%)	27 (73.0%)	
≥40 years	50 (43.9%)	4 (17.4%)	10 (27.0%)	
Conditioning, *n* (%)				<0.001
RIC	34 (29.8%)	0 (0.0%)	2 (5.4%)	
MAC	61 (53.5%)	23 (100.0%)	32 (86.5%)	
NMA	19 (16.7%)	0 (0.0%)	3 (8.1%)	
The first day post-transplant when a total neutrophil count > 0.5	21 (18–24)	21 (18–25)	15 (13–16)	<0.001
Acute GvHD, *n* (%)	40 (35.1%)	5 (21.7%)	22 (59.5%)	0.006
1 or 2 grade of aGvHD, *n* (%)	34 (29.8%)	3 (13%)	14 (37.8%)	0.110
3 or 4 grade of aGvHD, *n* (%)	6 (5.3%)	2 (8.7%)	8 (21.6%)	0.014
Median time of onset of acute GvHD, days (IQR)	41 (25–69)	27 (8–36)	18 (17–25)	0.001
Chronic GvHD, *n* (%)	28 (24.6%)	0 (0.0%)	4 (10.8%)	0.009
CMV ≥ 250 copies before treatment, *n* (%)	54 (47.4%)	8 (34.8%)	25 (67.6%)	0.030
CMV < 250 copies before treatment, *n* (%)	13 (11.4%)	0 (0.0%)	11 (29.7%)	0.002
Number of deceased patients until the end of observation, *n* (%)	46 (40.4%)	4 (17.4%)	28 (75.7%)	<0.001
Death due to infection, *n* (%)	19 (45.2%)	1 (25.0%)	14 (58.3%)	0.369
Death due to GvHD, *n* (%)	5 (11.9%)	0 (0.0%)	2 (8.3%)	0.709
Death due to relapse, *n* (%)	7 (16.7%)	1 (25.0%)	7 (29.2%)	0.484
Death from another or unknown cause, *n* (%)	15 (13.2%)	2 (50.0%)	1 (4.2%)	0.828
3-years overall survival S (t = 3)	49.1%	73.6%	33.4%	0.032
Median survival function	28 months	>12.6 months	10 months	

AML—acute myeloblastic leukemia, MDS—myelodysplastic syndromes, ALL—acute lymphoblastic leukemia, HL—Hodgkin Lymphoma, NHL—Non-Hodgkin Lymphoma, MM—multiple myeloma, OMF—myelofibrosis, CML—chronic myeloid leukemia, SAA—sever aplastic anemia; RIC—reduced intensity conditioning, MAC—myeloablative conditioning, NMA—non myeloabaltive conditioning; aGvHD—acute graft versus host disease, CMV—cytomegalovirus.

**Table 2:** Results of univariate and multivariate logistic regression concerning risk factors for aGvHD (in red: *p* < 0.05).

Risk Factor for aGvHD	*b*	*p*	*beta*	*p*	OR (95% CI)
PTCy-MMUD [yes]	−0.919	0.086	−1.835	0.088	0.16 (0.02–1.32)
ATG-MMUD [yes]	1.098	0.004	1.444	0.002	4.24 (1.73–10.4)
Donor age ≥ 31 years [yes]	1.163	0.001	0.057	<0.001	4.34 (1.98–9.49)

*b*—logistic regression coefficient in univariate analysis, *beta*—logistic regression coefficient in multivariate analysis, OR—odds ratio. aGvHD—acute graft versus host disease.

**Table 3:** Results of univariate and multivariate logistic regression concerning risk factors for cGvHD (in red: *p* < 0.05).

Risk Factor for cGvHD	*b*	*p*	*beta*	*p*	OR (95% CI)
PTCy-haplo [yes]	1.517	0.008	1.316	0.023	3.73 (1.20–11.6)
Donor: mismatched MUD [yes]	−1.517	0.008	−0.978	0.340	0.38 (0.05–2.87)
Donor age ≥ 42 years	1.423	0.001	1.265	0.003	3.54 (1.56–8.06)

cGvHD—chronic graft versus host disease.

## Data Availability

The authors confirm that the data supporting the findings of this study are available within the article.
